# *Jiawei Foshou San* Induces Apoptosis in Ectopic Endometrium Based on Systems Pharmacology, Molecular Docking, and Experimental Evidence

**DOI:** 10.1155/2019/2360367

**Published:** 2019-10-27

**Authors:** Jiahui Wei, Binxin Zhao, Chengling Zhang, Bingbing Shen, Ying Zhang, Changxi Li, Yi Chen

**Affiliations:** ^1^College of Pharmaceutical Sciences & Chinese Medicine, Southwest University, Chongqing, China; ^2^Chongqing Key Laboratory of New Drug Screening from Traditional Chinese Medicine, Chongqing, China; ^3^Pharmacology of Chinese Materia Medica—the Key Discipline Constructed by the State Administration of Traditional, Chinese Medicine, Chongqing, China; ^4^National Demonstration Center for Experimental Pharmacy Education (Southwest University), Chongqing, China; ^5^The First Affiliated Hospital to Army Medical University, Chongqing 400038, China; ^6^Department of Anesthesia, Chongqing Public Health Medical Center, Chongqing, China

## Abstract

*Foshou San* is a typical gynaecological formula with wild usage in traditional Chinese medicine. *Jiawei Foshou San* (JFS) is a novel ingredient prescription from *Foshou San* with antiendometriosis effect in unclear mechanisms. To uncover the potential application and proapoptotic mechanisms of JFS, JFS ingredient-drug target-disease networks, GO enrichment, and pathway analysis were established for potential application prediction. Molecular docking and validation *in vivo* were investigated by the proapoptotic mechanisms of JFS. In this study, 99 common targets were related to 108 diseases. 484 biological processes, 44 cell components, 59 molecular functions, and 37 pathways were significantly identified in GO enrichment and pathway analysis. In molecular docking, ligustrazine showed binding activity with Bcl-2, Bax, caspase-9, caspase-3, and PARP. In vivo, JFS elevated the shrink rate of ectopic endometrium, by suppressing E2 and PROG. An in-depth study showed that apoptosis was activated through diminishing Bcl-2, rising Bax and Bad, and expressing more caspase-3 and caspase-9 using JFS. JFS promoted the protein level of cleaved-PARP. In brief, JFS might be applied for various diseases through multiple targets and pathways, especially endometriosis by Bcl-2 pathway. These findings reveal the potential application for further evaluation and uncover the proapoptotic mechanism of JFS.

## 1. Introduction

Blood stasis syndrome in traditional Chinese medicine (TCM) is considered appearing in various chronic diseases, such as cardiocerebrovascular diseases, gynaecological diseases, tumor, and infectious diseases [1–3]. *Huoxue Huayu* recipe is regarded to ameliorate the blood stasis syndrome through activating blood and dissolving stasis in TCM [1, 4]. *Foshou San* formula is a classic *Huoxue Huayu* recipe, utilized extensively in gynaecological diseases, cardiocerebrovascular diseases, and hepatobiliary disease [5]. Two main active ingredients of *Foshou San*, ligustrazine and ferulic acid, are coordinated with tetrahydropalmatine to form *Jiawei Foshou San* (JFS). Our previous research has found that JFS has a good therapeutic effect on endometriosis (EMS), including reducing the inflammatory response and antimetastasis [3, 6]. However, the proapoptotic mechanism of JFS has not been measured in EMS. At the same time, it is worth to detect the potential application of JFS on disease belonging to blood stasis syndrome.

Systems pharmacology is a novel research field combined with pharmacology and systems biology-based technologies. Multicomponent and multitarget therapeutics and potential treatment of complex diseases are usually considered as the characteristics of TCM formulas. Thus, application of systems pharmacology in TCM will be helpful to uncover the interactions between components, targets, and pathways [7, 8]. Because not all targets are efficient therapeutic targets, systems pharmacology combined with FDA drug target database might be an efficient and promising way to broaden the potential therapeutic range of TCM [9].

EMS, one of the high-incidence gynaecological diseases, is defined as the presence of the active endometrial tissues at extrauterine sites [10, 11]. Currently, the pathogenesis of EMS is still unclear. Apoptosis is a physiologic process of programmed cell degeneration and necrosis under the action of apoptotic stimuli. It has been shown previously that abnormal apoptosis of endometrial cells is closely related to the occurrence and development of EMS [12, 13].

**Figure 1:**
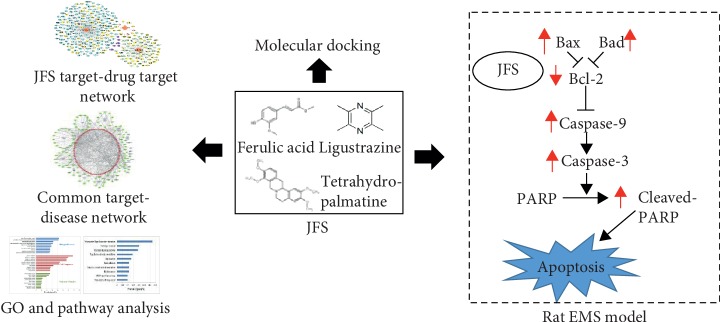
The flowchart of this study based on an integration of network pharmacology, molecular docking, and experimental evidence. JFS, *Jiawei Foshou San*; GO, Gene Ontology; EMS, endometriosis.

In this study, systems pharmacology was employed to establish JFS target-drug target and common target-disease networks. Then, the common targets were analysed for GO enrichment and pathway analysis. Binding activity of components and apoptosis-related targets was predicted by molecular docking. The proapoptosis of JFS was investigated through Bcl-2 pathway in the EMS rat model (Figure 1).

## 2. Materials and Methods

### 2.1. Networks Construction, GO Enrichment, and Pathway Analysis

TCMSP (http://lsp.nwu.edu.cn/tcmsp.php), SEA (http://sea.bkslab.org/), and BATMAN-TCM (http://bionet.ncpsb.org/batman-tcm/) databases were applied for potential targets of JFS compounds with the limitation of prediction score >80 and *P* > 0.05 [14]. DrugBank (https://www.drugbank.ca/drugs) and TTD (https://db.idrblab.org/ttd/) database were used to download pharmacological drug targets approved by the FDA. Common targets were screened by comparing JFS targets with FDA-approved drug targets. The connection between common targets and diseases was obtained from TTD and TCMSP. The diseases were classified according to MeSH (http://www.nlm.nih.gov/mesh/). Cytoscape 3.7.0 (http://www.cytoscape.org/) was used to generate the JFS target-drug target and common target-disease networks. Common targets were subjected to DAVID (https://david-d.ncifcrf.gov/) for GO enrichment and pathway analysis.

### 2.2. Molecular Docking

In SystemsDock Website (http://systemsdock.unit.oist.jp/iddp/home/index), molecular docking was analysed between JFS components with apoptosis-related targets. It shows certain binding activity with docking score >4.25, better binding activity with docking score >5.0, and strong binding activity with docking score >7.0 [15].

### 2.3. Reagents and Animals

The purity of ferulic acid (batch number: ZL2016061382), ligustrazine (batch number: ZL2016030815), and tetrahydropalmatine (batch number: ZL2016051235) were 99.8%, 99.3%, and 98.1%, respectively, from Nanjing Zelang Medical Technology (Nanjing, China). They were dispersed in 0.5% CMC-N with a ratio of 10 : 5:3. Gestrinone was purchased from Zizhu Pharmaceutical Co., Ltd. (Beijing, China). Rat PROG (CK-E30608R) and E2 ELISA Kit (CK-E30580R) were purchased from Yuanye Biomart (Shanghai, China). Primers for qPCR were designed by Beijing Dingguo Changsheng Biotechnology Co., Ltd. (Beijing, China). PrimeScript™ RT Reagent Kit was supplied by TaKaRa Bio Inc (Japan). SYBR™ Green Master Mix (A25741) was obtained from Thermo Fisher Scientific (USA). TUNEL Apoptosis Detection Kit (A113-01) was provided by Vazyme (Nanjing, China). DAPI solution (C02-04002) was purchased from Bioss (Beijing, China). Female Sprague Dawley rats weighing 180–220 g were supplied by Experimental Animals Institute of Chongqing Academy of Chinese Materia (Certification no. SCXK [yu] 2017-0003). They were housed in the Experimental Center, Southwest University. The study was conducted in accordance with the recommendations in the Guide for the Care and Use of Laboratory Animals of Southwest University (Approval no. 0002183). To minimize suffering, anesthesia with chloral hydrate (350 mg·kg^−1^) was administered intraperitoneally in surgical procedure and blood collection.

### 2.4. EMS Rat Model and Treatment

According to the previous study, 71 estrous rats were autotransplanted of self-uterine to establish the EMS model [3]. The right uterine was divided into 5 × 5 mm pieces for transplantation to the left peritoneum. Volume formula of endometrial autografts was calculated with 0.52 × length × width × height by vernier caliper after 28 days [16]. Endometrial autografts, larger than 20 mm^3^ with surface blood, were regarded as the successful EMS models. Then, EMS rats were randomly separated into 4 groups according to the volume of endometrial autografts, EMS group with 0.5% CMC-Na, 45, 90, 180 mg·kg^−1^ JFS groups, and 0.5 mg·kg^−1^ gestrinone group. After chronically administered for 28 days, the volume change rate was calculated with the formula (volume after treatment−volume pretreatment)/volume pretreatment × 100%.

### 2.5. Detection of E2 and PROG in Serum by ELISA

Serum of abdominal aorta was prepared for analysis. Following ELISA kit instruction, the equilibrated plates were added with serum samples and dilution reagents. The HRP-conjugated reagent was put into each well, which was covered with adhesive strips. After washing, chromogenic and stop solutions were inputted step by step. Absorbance readings at 450 nm were probed with ELX800 Universal Microplate Reader (Bio-Tek Instruments, USA).

### 2.6. TUNEL Assay

TUNEL assay was performed according to the manufacturer's protocol. In brief, after dewaxed and hydrated, the tissue sections were incubated with proteinase K for 20 min at room temperature. After incubated with equilibration buffer, sections were covered with TUNEL reaction mixture for 1 h at 37°C. Finally, the sections were washed in PBS three times and counterstained with DAPI. The sections were examined and photographed under fluorescence microscopy (DFC310 FX, Leica, Germany). Apoptosis index (AI) = apoptotic cell number/total cell number × 100% [17].

### 2.7. Real-Time PCR

Total RNA was extracted from ectopic endometrium using TRIzol reagent (Invitrogen, CA, USA) following the process described previously [18]. The synthesis of cDNA was performed with PrimeScript™ RT Reagent Kit. The mRNA levels were detected using SYBR™ Green Master Mix with CFX96 Real-Time System (Bio-Rad, USA). The relative expression of target genes was calculated by the 2-∆∆CT method using primer sequences (Table 1). The control reference gene was GAPDH.

### 2.8. Western Blot Analysis

Protein lysates of ectopic endometrium were separated by 10 or 12% SDS-PAGE and transferred to polyvinylidene difluoride membranes (Millipore, USA). The membranes were blocked with 5% skim milk in TBST for 2 hours. The membranes were incubated overnight at 4°C with rabbit anti-Bcl-2, rabbit anti-Bax (1 : 500 dilution; Proteintech Biotechnology, Wuhan, China), rabbit anti-PARP (1 : 250 dilution; Biosynthesis Biotechnology, Beijing, China), rabbit anti-cleaved-PARP (1 : 1000 dilution; Cell Signaling Technology, USA), and rabbit anti-*β*-actin (1 : 5000 dilution; Proteintech Biotechnology, Wuhan, China). After washing, the membranes were probed with HRP-conjugated goat anti-rabbit secondary antibody (1 : 1000 dilution; Multi Sciences, Hangzhou, China). The blots were analysed with the Tanon 5200 imaging system (Tanon, China) with *β*-actin as an internal control.

### 2.9. Statistical Analysis

All data were presented as mean ± SD and compared by one-way ANOVA test using SPSS software (version 21). *P* < 0.05 was regarded as statistically significant difference.

## 3. Results

### 3.1. JFS Target-Drug Target Network Construction and Analysis

**Figure 2:**
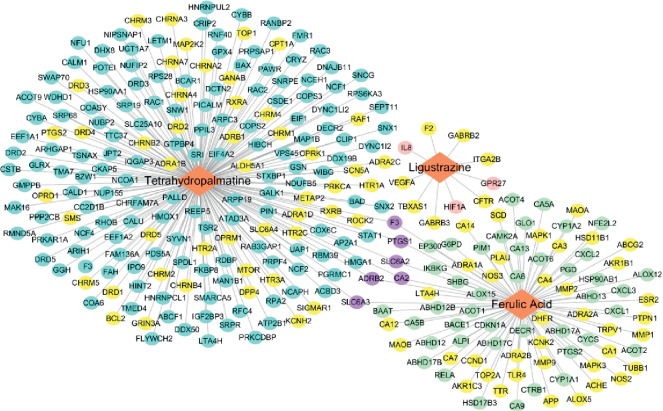
JFS target-drug target network. Blue circles, pink circles, and green circles represent the targets of tetrahydropalmatine, ligustrazine, and ferulic acid. Yellow circles represent 93 common targets between JFS targets and FDA-approved drug targets. Purple circles represent the common targets for ferulic acid, tetrahydropalmatine, and FDA-approved drug targets. JFS, *Jiawei Foshou San*.

Through TCMSP, SEA, and BATMAN-TCM databases, there were 293 potential targets corresponding to JFS ingredients, including 10 for ligustrazine, 86 for ferulic acid, and 197 for tetrahydropalmatine ([Supplementary-material supplementary-material-1]). 1215 FDA-approved drug targets were obtained from DrugBank and TTD databases ([Supplementary-material supplementary-material-1]). JFS target-drug target network consisted of 290 nodes and 293 edges (Figure 2). 99 common targets were shifted from the network, 7 in ligustrazine, 44 in ferulic acid, and 54 in tetrahydropalmatine, including Bcl-2 ([Supplementary-material supplementary-material-1]). Interestingly, those 6 targets were belonged to not only ferulic acid and tetrahydropalmatine targets, but also FDA-approved drug targets, such as ADRB2, CA2, F3, PTGS1, SLC6A2, and SLC6A3. It was suggested that the therapeutic properties of JFS might be based on these targets.

### 3.2. Common Target-Disease Network Construction and Analysis

**Figure 3:**
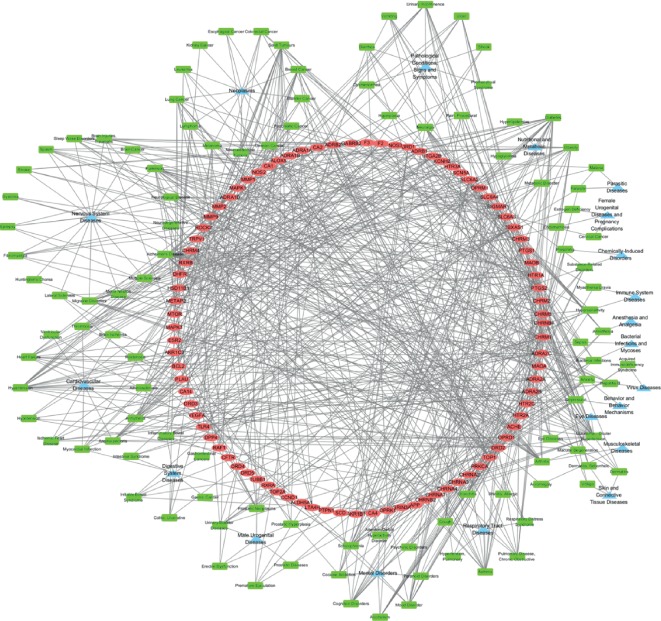
Common target-disease network. Red ellipses represent 99 common targets, and green rectangles represent diseases. 108 diseases were classified into 30 categories according to the MeSH database. MeSH, Medical Subject Headings.

The common target-disease network consisted of 216 nodes and 766 edges. 99 common targets were related to 108 diseases of 30 groups (Figure 3, [Supplementary-material supplementary-material-1]). A large proportion of common targets were distributed in nervous system diseases (15.7%), neoplasms (11.1%), cardiovascular diseases (11.1%), and pathological conditions, signs and symptoms (9.3%). Among these diseases, female urogenital diseases and pregnancy complications contained 2.8% targets, mainly including EMS, estrogen deficiency, and cervical cancer. It was worthwhile to note that PTGS2 and MTOR were related to EMS. This suggests that JFS might have potential therapeutic effects on these diseases.

### 3.3. GO Enrichment and Pathway Analysis

**Figure 4:**
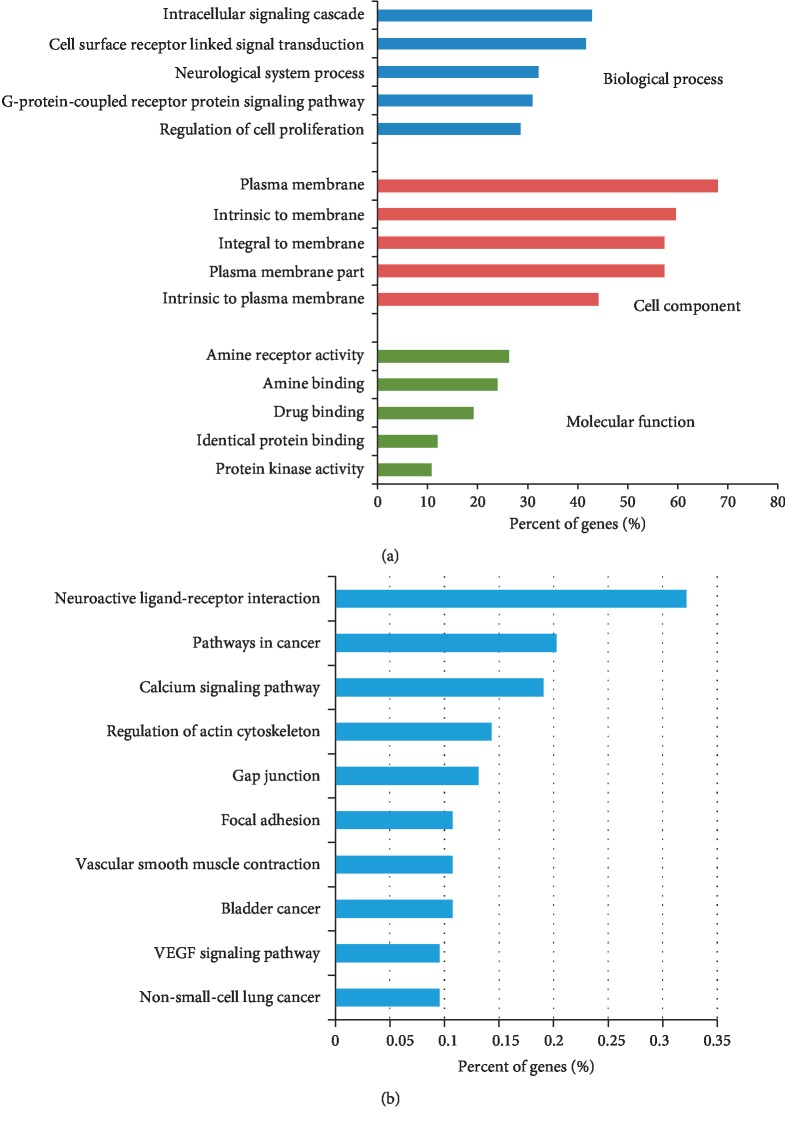
GO enrichment and pathway analysis of common targets. (a) Top 5 significantly enriched terms are shown in the biological process, cell component, and molecular function from DAVID. (b) The top 10 remarkably enriched pathways are shown.

In GO enrichment, 484 of 584 biological processes, 59 of 81 molecular functions, and 44 of 50 cell components were significantly identified in common targets from DAVID (*P* < 0.05) ([Supplementary-material supplementary-material-1]). The top 5 remarkably enriched terms are listed in Figure 4(a). According to pathway analysis, 37 common target-related pathways were identified significantly in DAVID. The top 10 representative pathways were exhibited (Figure 4(b)). Bcl-2 was mainly distributed in 6 pathways, which were small-cell lung cancer, prostate cancer, pathways in cancer, neurotrophin signaling pathway, focal adhesion, and colorectal cancer ([Supplementary-material supplementary-material-1]).

### 3.4. Binding Activity of JFS with Apoptosis-Related Targets

Descent of apoptosis in ectopic endometria is considered as the principal mechanism of EMS [19]. In Figure 2, Bcl-2 was one of the common targets between tetrahydropalmatine and drug targets. Consequently, apoptosis-related targets, including Bcl-2, Bax, Bad, caspase-3, caspase-9, and PARP, were prepared for molecular docking. Ligustrazine showed the binding activity with 5 targets, a certain binding with caspase-9 (docking score >4.25) and the better binding with Bcl-2, Bax, and caspase-3, and PARP (docking score >5.0). Ferulic acid and tetrahydropalmatine had no obvious binding with 6 targets (Table 2). This indicates that ligustrazine in JFS may have a primary effect on apoptosis.

### 3.5. JFS Elevated the Reduction Rate of Volume in EMS

**Figure 5:**
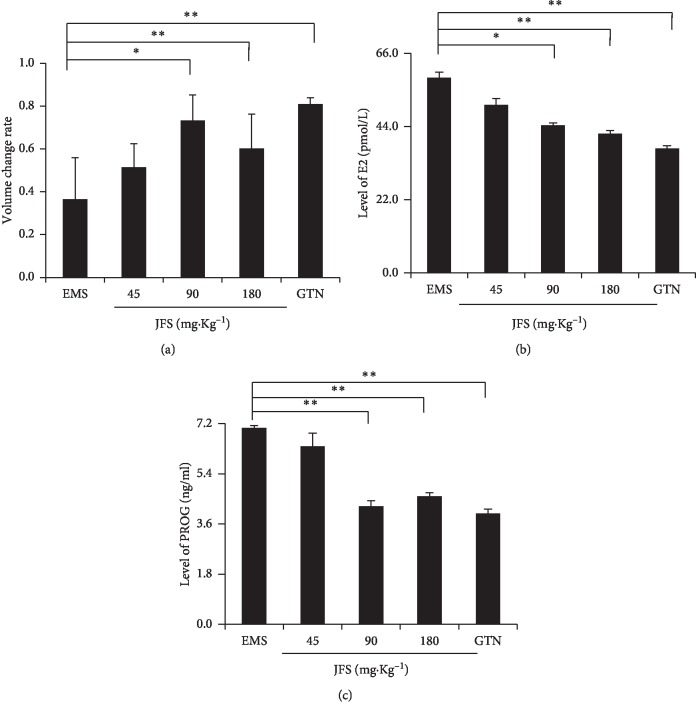
Regulation of JFS on volume change rate of ectopic endometrium and the levels of E2 and PROG. (a) The volume change rates of ectopic endometrium were calculated after administration for 28 days. (b)-(c) Serum levels of E2 and PROG were detected by ELISA in different groups. ^*∗*^*P* < 0.05, ^*∗∗*^*P* < 0.01 compared with the EMS group. Columns, mean (*n* = 6). Bars, SD. EMS, endometriosis; JFS, *Jiawei Foshou San*; GTN, gestrinone.

28 days after surgery, EMS model was successfully demonstrated in 56 rats. The success rate of the model was 79%. Difference in the ectopic endometrium volume was not obvious between 5 groups before administration. After treatment for 28 days, the volume change rate was measured in different groups compared with pretreatment. In the EMS group, the volume of ectopic endometrium was slightly reduced. The volume change rates of 90 and 180 mg·kg^−1^ JFS groups were obviously higher compared with the EMS group (*P* < 0.05) (Figure 5(a)). This suggests that JFS had obvious inhibitory effects on transplant growth in a dose-dependent manner.

### 3.6. JFS Degraded E2 and PROG Levels

EMS is considered as an estrogen-dependent disease, accompanied by perturbations in progesterone signal [20]. In the previous study, the therapeutic medicine for endometriosis has been based upon systemic hormonal alteration. So E2 and PROG are considered as the indicators of therapy [21, 22]. The ELISA results showed that high serum levels of E2 and PROG were observed in the EMS group. The E2 and PROG levels in the JFS groups decreased compared with the EMS group, especially in 90 and 180 mg kg^−1^ JFS groups (*P* < 0.05) (Figures 5(b) and 5(c)).

### 3.7. JFS Promoted Apoptosis in Ectopic Endometrial Tissues

**Figure 6:**
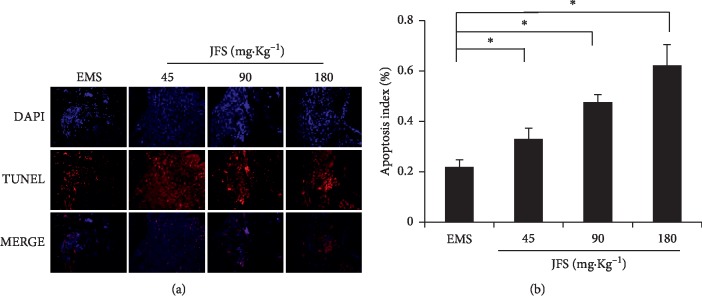
Detection of apoptosis in ectopic endometrial tissues. (a) Apoptosis in ectopic endometria of different groups was observed by TUNEL assay. DAPI-stained nuclei appeared in blue. Red-stained tissue appeared in red due to the presence of apoptotic cells. (b) The apoptotic index of ectopic endometrial tissues. ^*∗*^*P* < 0.05 compared to the EMS group. Columns, mean (*n* = 3). Bars, SD. Magnification, ×200. EMS, endometriosis; JFS, *Jiawei Foshou San*.

The red fluorescence intensity and apoptotic cells' number increased with the treatment of JFS (Figure 6(a)). Meanwhile, compared with the EMS group, the AI in endometrial tissues was obviously promoted by JFS, especially in 180 mg·kg^−1^ JFS (Figure 6(b)). This suggests that JFS had induced apoptosis in ectopic endometrial tissues in a dose-dependent manner.

### 3.8. Activation of Bcl-2 Pathway by JFS

**Figure 7:**
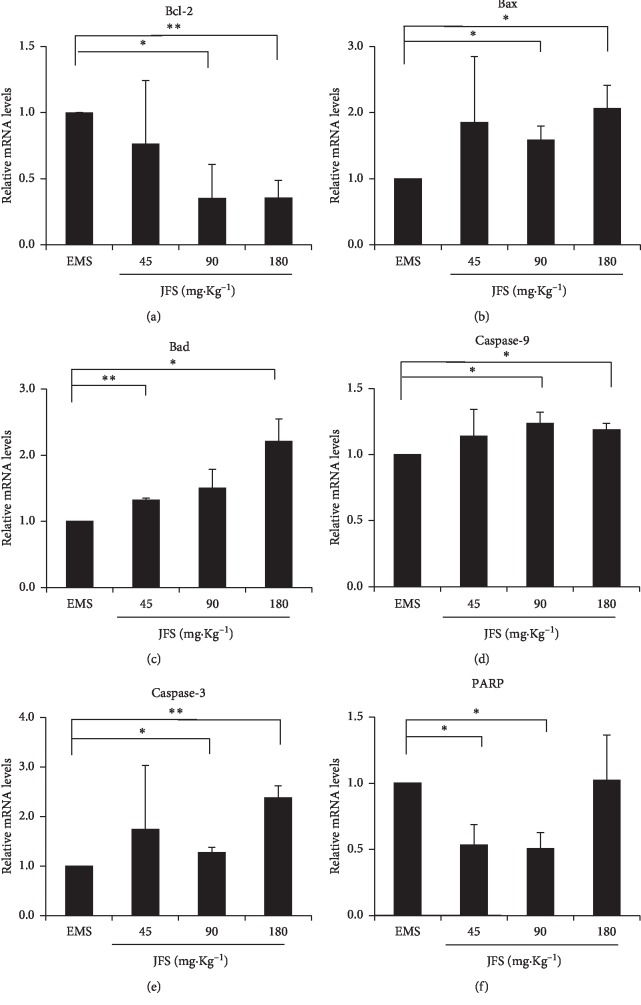
Gene expression of Bcl-2 pathway by JFS. (a)–(f) The mRNA levels of Bcl-2, Bax, Bad, caspase-3, caspase-9, and PARP were detected by qPCR in different groups. ^*∗*^*P* < 0.05 compared to the EMS group. Columns, mean (*n* = 3). Bars, SD. EMS, endometriosis; JFS, *Jiawei Foshou San*.

Using JFS for 28 days, genes and proteins of Bcl-2 pathway were detected, including Bcl-2, Bax, Bad, caspase-3, caspase-9, and PARP. JFS decreased the mRNA level of Bcl-2 by raising the mRNA levels of Bax and Bad (Figures 7(a), 7(b), and 7(c)). JFS obviously upregulated the gene expression of caspase-9 and caspase-3 compared to the EMS group (*P* < 0.05) (Figures 7(d) and 7(e)). PARP mRNA level reduced in 45 and 90 mg·kg^−1^ JFS groups (Figure 7(f)).

**Figure 8:**
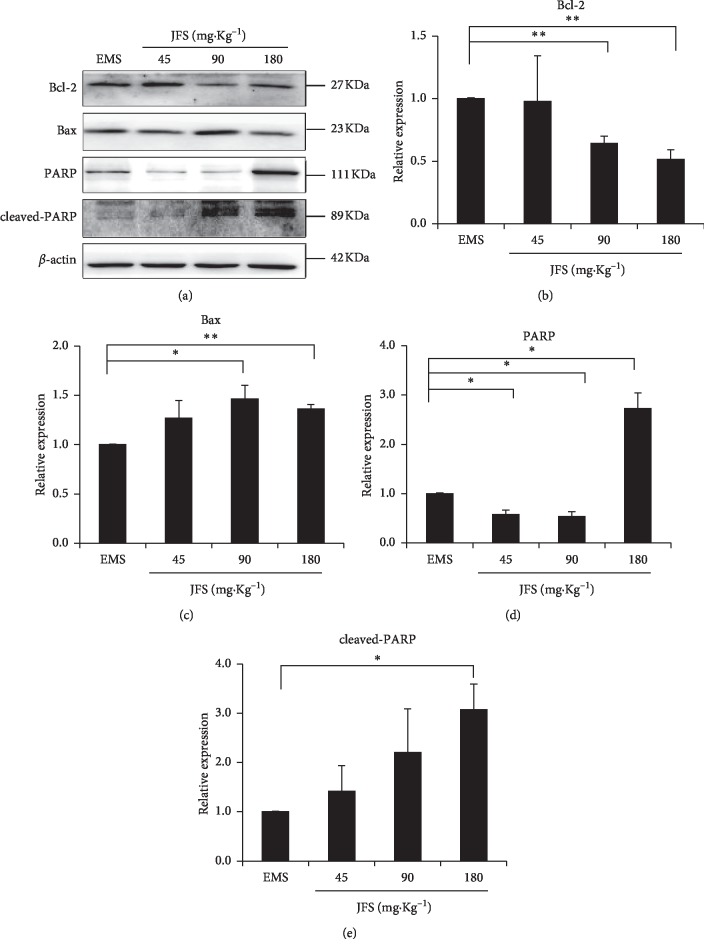
Protein levels of Bcl-2 pathway treated with JFS. (a)–(e) The protein levels of Bcl-2, Bax, PARP, and cleaved-PARP were detected by western blotting, and the ratios of Bcl-2, Bax, PARP, and cleaved-PARP to *β*-actin are shown. ^*∗*^*P* < 0.05 compared to EMS, ^*∗∗*^*P* < 0.01 compared to EMS. Columns, mean (*n* = 3). Bars, SD. EMS, endometriosis; JFS, *Jiawei Foshou San*.

Furthermore, the protein expression was in accordance with gene expression. The protein level of Bcl-2 attenuated in 90 and 180 mg·kg^−1^ JFS groups, accompanied with accumulation of Bax (Figures 8(a), 8(b), and 8(c)). PARP protein decreased significantly in 45 and 90 mg·kg^−1^ JFS groups and however increased in 180 mg·kg^−1^ JFS group. It was noteworthy that the protein expression of cleaved-PARP remarkably enhanced in 180 mg·kg^−1^ JFS group compared with the EMS group (*P* < 0.05) (Figures 8(a), 8(d), and 8(e)). This result suggests that the activation of apoptosis by JFS was connected with promotion of Bcl-2 pathway.

## 4. Discussion

Systems pharmacology is an ideal method to study the interaction between multicomponents, multitargets, and different pathways from online databases. Nevertheless, not all compound targets are pharmacological drug targets. So it will be more efficient to combine the systems pharmacology databases with therapeutic FDA-approved drug targets. Following this screening strategy, PTGS2 and MTOR were discovered as EMS drug targets associated with tetrahydropalmatine, one of the JFS components. Although ligustrazine and ferulic acid suppressed PTGS2 and MTOR in tumor or nervous system diseases [23–27], the cooperation of ligustrazine, ferulic acid, and tetrahydropalmatine is unknown whether or not via PTGS2 or MTOR, in spite of their valid effect in EMS. It needs further research. At the same time, it was worthwhile to note that 6 common targets were inspected among ferulic acid, tetrahydropalmatine, and drug targets, which comprise ADRB2, CA2, F3, PTGS1, SLC6A2, and SLC6A3. ADRB2 encodes beta-2-adrenergic receptor which mainly binds epinephrine. It is reported that activation of ADRB2 promotes EMS development by stress and surgery [28–30]. In cancer and cardiomyocytes, ADRB2 inhibits apoptosis mainly through Bcl-2/Bax/caspase-9 pathway [31–35]. Herein, ADRB2 might be the therapeutic target in EMS. We should try to use ADRB2 inhibitor in EMS or screen TCM drugs on ADRB2.

In JFS target-drug target network, Bcl-2 was the target of tetrahydropalmatine. But in molecular docking, docking score between tetrahydropalmatine and Bcl-2 was 3.709, and docking score between ligustrazine and Bcl-2 was 5.938. Those implied that ligustrazine not tetrahydropalmatine showed the binding with Bcl-2. In recent years, ligustrazine and tetrahydropalmatine have been found different effect on apoptosis through Bcl-2 pathway. On the one hand, tetrahydropalmatine reduces the apoptosis through enhancing the expression of Bcl-2 and reducing Bax/Bcl-2 ratio in cerebral ischemia-reperfusion and endothelial cells against *γ*-irradiation injury [36–38]. On the other hand, levo-tetrahydropalmatine induces apoptosis through upregulating Bcl-2 in hepatocytes [39]. Meanwhile, ligustrazine exerts the opposite regulation of Bcl-2, including inhibiting apoptosis in acute myocardial ischemia and neuron cells, triggering apoptosis in cancer [40–43]. Basically, the perplexing effect of ligustrazine and tetrahydropalmatine on apoptosis might relate to the concentration and pathological environments in various diseases.

Bcl-2 is a well-known regulator inhibiting cellular apoptosis, while Bax and Bad trigger the cascades leading to apoptosis. Downstream caspase-9 causes activation of caspase-3, which induces PARP to cleavage PARP [44]. In this study, JFS elevated apoptosis of ectopic endometrium using the TUNEL assay in the EMS rat model. In addition, the gene and protein expression of Bcl-2 diminished with rising Bax and Bad by JFS. Caspase-9 and caspase-3 genes expressed much more than the EMS group. JFS promoted the protein level of cleaved-PARP. It is noticeable that 180 mg·kg^−1^ JFS enhanced the PARP, while increasing cleaved-PARP. In the previous study, JFS ingredients have different effects on PARP, tetrahydropalmatine, and ferulic acid attenuating it [38, 45–49] and ligustrazine promoting it [50]. So it might be the foundation of the synergistic therapeutic effect of JFS ingredients. The above data suggest that apoptotic promotion of JFS may be related to Bcl-2 pathway in EMS. But in the future, the effect of JFS on other apoptosis factors such as P53, cyt C, caspase-6, and caspase-7 needs further investigation.

## 5. Conclusions

In this study, using systems pharmacology databases, the connection was uncovered between JFS ingredients, JFS targets, FDA-approved drug targets, disease, and pathways. In molecular docking, JFS ingredients were bound to apoptosis-related targets. JFS promoted apoptosis through activation of Bcl-2 pathway in EMS.

## Figures and Tables

**Table 1:** Sequences of primers.

Primer name	Sequences (5′-3′)
Bcl-2-F	GGTGAACTGGGGGAGGATTG
Bcl-2-R	AGAGCGATGTTGTCCACCAG
Bax-F	AAGAAGCTGAGCGAGTGTCT
Bax-R	CAAAGATGGTCACTGTCTGC
Bad-F	CCGAAGAATGAGCGATGAAT
Bad-R	GATAATGCGCGTCCAACTG
Caspase-9-F	AGCTGGCCCAGTGTGAATAC
Caspase-9-R	GCTCCCACCTCAGTCAACTC
Caspase-3-F	TGTATGCTTACTCTACCGCACCCG
Caspase-3-R	GCGCAAAGTGACTGGATGAACC
PARP-F	CCAGCAGAAGGTCAAGAAGAC
PARP-R	ACCTCCATGCTGGCCTTT
GAPDH-F	AGACAGCCGCATCTTCTTGT
GAPDH-R	CTTGCCGTGGGTAGAGTCAT

**Table 2:** Docking scores of JFS with apoptosis-related targets.

Compound	Target	PDB ID	Docking score
Ligustrazine	Bax	2G5B	6.859
	PARP	2RIQ	6.641
	Bcl-2	2W3L	5.938
	Caspase-3	2DKO	5.105
	Caspase-9	3D9T	4.66
	Bad	2BZW	—

Ferulic acid	Caspase-9	3D9T	4.004
	Caspase-3	2DKO	3.552
	Bcl-2	2W3L	3.379
	Bax	2G5B	3.146
	PARP	2RIQ	3.05
	Bad	2BZW	—

Tetrahydropalmatine	Caspase-9	3D9T	3.84
	Caspase-3	2DKO	3.755
	PARP	2RIQ	3.745
	Bcl-2	2W3L	3.709
	Bax	2G5B	3.701
	Bad	2BZW	—

## Data Availability

The network, GO, and pathway analysis data used to support the findings of this study are included within the supplementary information files. The other data used to support the findings of this study are available from the corresponding author upon request.

## Supplementary Materials

Supplementary Materials Table S1: detailed information on targets for JFS compounds. Table S2: detailed information on pharmacological targets of FDA-approved drugs. Table S3: 99 common targets between JFS targets and FDA-approved drug targets. Table S4: detailed information on common target-diseases and disease classification. Table S5: GO analysis and KEGG pathway enrichment of 99 common targets from DAVID.
